# Intranasal Application of Peptides Modulating the Neuropeptide Y System

**DOI:** 10.1021/acsptsci.5c00082

**Published:** 2025-04-01

**Authors:** Eva-Maria Jülke, Benginur Özbay, Marcin Nowicki, Sylvia Els-Heindl, Kerstin Immig, Karin Mörl, Ingo Bechmann, Annette G. Beck-Sickinger

**Affiliations:** † Institute of Biochemistry, Faculty of Life Sciences, 9180 Leipzig University, Brüderstr. 34, 04103 Leipzig, Germany; ‡ Institute of Anatomy, Faculty of Medicine, Leipzig University, Liebigstraße 13, 04103 Leipzig, Germany

**Keywords:** neuropeptide Y, peptide YY, neuropeptide Y receptors, peptide therapeutics, nasal application

## Abstract

The neuropeptide Y multireceptor–multiligand system plays an important role in multiple physiological processes. Targeting the neuropeptide Y_1_ (Y_1_R) and Y_2_ (Y_2_R) receptors has gained interest in treating weight and mental disorders. Nose-to-brain delivery is an effective tool to overcome the challenges of peptide delivery to cerebral structures. In this study, fluorescently labeled peptides that selectively activate either Y_1_R or Y_2_R were studied. The permeability of these compounds was evaluated on Calu-3 cells, a model system of the nasal mucosa. Particular attention was paid to the stability of peptides, and translocation of the intact compounds was demonstrated by combining a permeability assay with a receptor activation assay. Two compounds, selectively targeting either Y_1_R or Y_2_R, were selected, and their uptake after intranasal application was analyzed *in vivo*. Two different imaging systems were compared: whole slide scanning and confocal microscopy. Both methods allow detecting specific signals from the fluorescently labeled peptides. While whole slide scanning provides a comprehensive anatomical overview, confocal microscopy offers an improved signal-to-noise ratio. Finally, peptide-specific signals were quantified over time, displaying rapid peptide uptake within the first 15 min and sustained signals for up to 24 h. Overall, cell-based and *in vivo* assays were combined to select peptides with high pharmacological potential for nasal applications.

## Graphical abstract

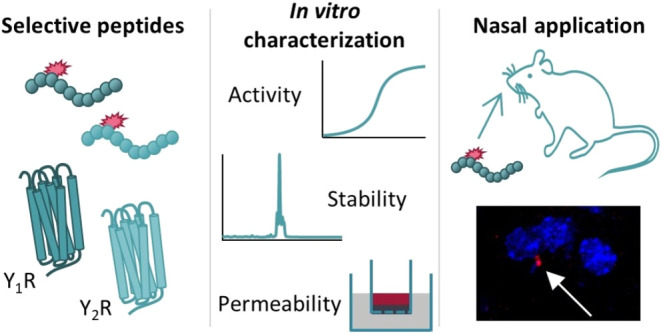

The neuropeptide Y (NPY) multireceptor–multiligand system is involved in various physiological functions including the regulation of energy homeostasis, stress and anxiety, cognitive functions, angiogenesis, osteogenesis, and the circadian rhythm.^1–8^ In humans, these biological effects are mediated by four neuropeptide Y receptors (YR), namely, Y_1_R, Y_2_R, Y_4_R, and Y_5_R. They are activated by the three peptide ligands: NPY, peptide YY (PYY), and pancreatic polypeptide (PP). The whole system has gained significant attention for the treatment of diseases such as obesity, cachexia, post-traumatic stress disorder (PTSD), depression, and cancer.^9–14^

Due to the high diversity of biological processes in which the NPY system is involved, selective targeting of neuropeptide Y receptors is required for successful therapeutic applications. In particular, selective activation of one of the highly conserved receptors is challenging to achieve with small molecules.^15^ In contrast, peptides provide an increased surface area that allows access to a greater number of interaction sites within the receptor-binding pocket, thereby facilitating the identification of selective compounds. Following this principle, selective, agonistic peptides have been described for all neuropeptide Y receptor subtypes.^16–22^ For example, while pNPY is highly active at both receptors, [F^7^, P^34^]-pNPY and [Ahx^5–24^]-pNPY (Ahx: 6-aminohexanoic acid) are selective agonists for Y_1_R and Y_2_R, respectively.^16,17^ Despite the high sequence similarity to pNPY (Table 1), PYY_3–36_ preferentially activates Y_2_R over Y_1_R due to the truncated *N*-terminus.^23^ *In vivo*, PYY_3–36_ originates from the cleavage of PYY by dipeptidyl aminopeptidase 4 (DPP-4), and the truncated peptide is more selective than the intact one.^21,24^

**Table 1:**
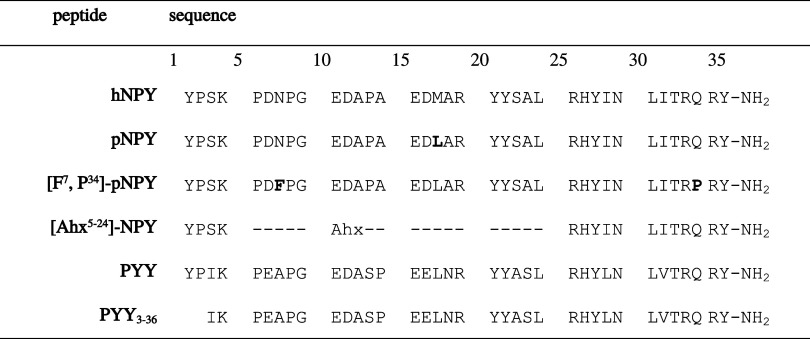
Peptide sequences^a^

a Abbreviations: Ahx, 6-aminohexanoic acid; hNPY, human neuropeptide Y; pNPY, porcine neuropeptide Y; PYY, peptide YY.

While peptides provide an advantageous platform for the development of highly potent and selective therapeutics, the delivery of these chemical structures, particularly to cerebral receptors, is challenging. Most peptides have low membrane permeability, resulting in low oral bioavailability and almost no penetration of the blood–brain barrier (BBB).^25^ Further, systemic distribution through the bloodstream can result in the rapid degradation of peptides by proteolytic cleavage and renal clearance. Intranasal delivery of peptides has a great potential to overcome these challenges. Direct nose-to-brain delivery allows for peptide entry from the olfactory epithelium and further translocation via olfactory or trigeminal neurons to the central nervous system.^26,27^

Calu-3 cells are used as the model system for the nasal mucosa. This cell line is derived from human bronchial adenocarcinoma and is a suitable, scalable, and low-cost model compared to primary cells. Upon differentiation, Calu-3 cells were found to form a robust cell layer including tight junctions and mucus secretion.^28–30^ While differentiation of Calu-3 cells can also be performed under liquid cover culture (LCC), differentiation on an air–liquid interface (ALI) is the preferred method for permeability studies because, among other relevant characteristics, the expression of transporter proteins was found to be increased in ALI cultures.^31^

In our study, we aimed to selectively target either the Y_1_R or Y_2_R receptor by nasal application of fluorescently labeled peptides in specific brain areas. The peptides were analyzed in detail using cell-based assays for receptor activation, stability, and permeability through Calu-3 cells. Peptide stability was found to be a critical aspect affecting the obtained data. While the Calu-3 cell model primarily reflects epithelial transport and does not fully capture neuronal uptake mechanisms, promising compounds were selected for *in vivo* application in mice. Uptake and cerebral delivery were tracked within the olfactory bulb, the cortex, and the hypothalamus using fluorescence imaging. Thereby, vesicle-like structures were observed, and the specific signals were quantified. While those most likely do not present the total amount of peptide taken up to the central nervous system, the obtained data provided interesting insights into the dynamics of nasal applications.

## Material and Methods

### Synthesis and Analysis of Peptides

Solid-phase peptide synthesis (SPPS) was carried out in a 15 μmol scale using the fluorenylmethoxycarbonyl (Fmoc)/*tert-*butyl (*t-*Bu) protection group strategy on NovaSyn TG R resin (Merck KGaA, Darmstadt, Germany), as described previously.^32–36^ Couplings were performed using a SYROI synthesis robot (MultiSynTech GmbH, Witten, Germany) in double-coupling procedures with 8 equiv of amino acid, 8 equiv of ethyl cyanohydroxyiminoacetate (Oxyma, Iris Biotech), and *N,N*-diisopropylcarbodiimide (DIC, Iris Biotech) in *N,N*-dimethylformamide (DMF, VWR, Radnor) and a reaction time of 42 min per cycle. Fmoc cleavage was performed in two subsequent cycles by applying 40% (v/v) and 20% (v/v) piperidine (Sigma-Aldrich, St. Louis, Missouri) in DMF for 3 and 10 min, respectively. For fluorescent labeling, 2 eqiv of 6-carboxytetramethylrhodamine (Tam, ChemPep Inc., Wellington) was dissolved with 1.9 eqiv. of hexafluorophosphate azabenzotriazole tetramethyl uronium (HATU, Novabiochem, Schwalbach, Germany) and 2 eqiv. of *N,N*-diisopropylethylamine (DIPEA, Carl Roth GmbH & Co. KG, Karlsruhe, Germany) in 500 μL of DMF and incubated twice for at least 3 h. Manual Fmoc deprotection was achieved by incubation in 20% (v/v) piperidine in DMF twice for 10 min. The monomethoxytrityl (Mmt) protection group was removed by incubating the resins 15 times in 2% (v/v) trifluoroacetic acid (TFA, Sigma-Aldrich) and 5% (v/v) triisopropyl silane (TIS, Merck) in dichloromethane (DCM) for 1 min, and 1-(4,4-dimethyl-2,6-dioxocyclohex-1-ylidene)­ethyl (Dde) was deprotected using 12 incubations in 2% (v/v) hydrazine (Sigma-Aldrich) in DMF for 10 min. For full cleavage, resins were incubated in 1 mL of 7% thioanisole (TA, Sigma-Aldrich) and 3% (v/v) ethane-1,2-dithiol (EDT, Sigma-Aldrich) in TFA for 3 h. Then, peptides were precipitated in 10 mL of diethyl ether (Merck) for at least 30 min at −20 °C and subsequently washed five times with ice-cold diethyl ether.

Preparative reversed-phase high-performance liquid chromatography (RP-HPLC, Nexera, Shimadzu, Kyo̅to, Japan) was performed on an Aeris Peptide 5 μm XB-C18 column (250 mm × 21.2 mm, Phenomenex, Torrance). A linear gradient of eluent B [0.08% (v/v) TFA in acetonitrile (ACN)] in eluent A [0.1% (v/v) TFA in H_2_O] with an increase of 1% eluent B/min at a flow rate of 15 mL/min was applied, and peptide elution was monitored from absorption at 220 nm. The purity of peptides was confirmed by analytical RP-HPLC (Hitachi Chromaster, VWR), and mass spectrometry was performed with an electrospray ionization (ESI)-orbitrap system (Thermo Fisher Scientific, Waltham, Massachusetts) or with matrix-assisted laser desorption ionization time-of-flight mass spectrometry (MALDI-ToF MS, MALDI-ToF-ToF Ultraflex III, or Microflex, Bruker, Billerica).

### IP_1_ Accumulation Assay

Receptor activity assays were performed with the HTRF IP-One Gq Detection Kit (Revvity, Waltham, Massachusetts, former Cisbio) in COS-7_hY_1_R_Gα_Δ6qi4myr_ and COS-7_hY_2_R_ Gα_Δ6qi4myr_ cells, as reported previously.^37–39^ Cells were cultured in Dulbecco’s modified Eagle’s medium (DMEM, Biowest, Nuaillé, France) supplemented with 10% heat-inactivated fetal bovine serum (FBS, Biochrom, Berlin, Germany), 133 μg/mL hygromycin B (Invivogen, Toulouse, France), and 1.5 mg/mL G418 sulfate (Invivogen) under a humidified atmosphere at 37 °C and 5% CO_2_. For the activity assay, 8000 cells were seeded in 20 μL per well in a white 364-well plate (Greiner Bio-One International GmbH, Kremsmünster, Austria) and cultured under standard conditions overnight. Cells were stimulated with 15 μL of peptide dilution series prepared from 1 mM dimethyl sulfoxide (DMSO, Sigma-Aldrich) stocks in Hanks’ balanced salt solution (HBSS, Merck) substituted with 10 mM lithium chloride (Sigma-Aldrich). After incubation in a humidified atmosphere for 45 min, 3 μL of each of the manufacturer’s antibody cryptate and inositol monophosphate (IP_1_) coupled to the d2 acceptor were added per well and incubated for 1 h at room temperature. Homogeneous time-resolved fluorescence (HTRF) signals were measured with a plate reader (Spark, Tecan Trading AG, Männedorf, Switzerland) and calculated by dividing the fluorescent signal of the Förster resonance energy transfer (FRET) acceptor (excitation wavelength (λ_ex_) = 320 ± 25 nm, emission wavelength (λ_em_) = 665 ± 8 nm, lag time = 100 μs) by the signal of the FRET donor (λ_ex_ = 320 ± 25 nm, λ_em_ = 620 ± 10 nm, lag time 100 μs). Data analysis was carried out with Prism (version 10, GraphPad Software, Boston, Massachusetts). Only concentration–response curves within 10–90% of the maximal effect of an IP_1_ standard curve were included. These signals were normalized to the control peptide, and the half-maximal efficient concentration (EC_50_) and maximal effect (*E*_max_) were calculated using a nonlinear regression curve fit.

The IP_1_ accumulation assay combined with the permeability assay was carried out similarly, but dilution series and samples from Calu-3 permeability assay at 0, 2, 4, and 6 h were mixed 1:1 (v/v) with HBSS substituted with 20 mM LiCl.

### Culture and Differentiation of Calu-3 Cells

Calu-3 cells were obtained from the American Type Culture Collection (American Type Culture Collection (ATCC), Manassas, Virginia) and used in passages 20–27. Minimum essential medium (MEM, Biowest) supplemented with 10% (v/v) heat-inactivated FBS, 1× nonessential amino acids (Gibco, Grand Island, New York), 1 mM sodium pyruvate (Gibco), and 2 mM glutamine (Gibco) was used as cell culture medium, and cells were kept in a humidified atmosphere. For detachment, cells were washed twice with Dulbecco’s phosphate-buffered saline (DPBS) and incubated in TrypLE Express Enzym (Gibco) for 15–30 min at 37 °C. Cells were resuspended in the cell culture medium and transferred to a new flask.

For differentiation, cells were seeded to a density of 200,000 cells/cm^2^ in Thincert inserts with a pore size of 0.4 μm (Greiner Bio-One) in cell culture medium supplemented with 50 μg/mL gentamicin (Gibco). After 3 days under a humidified atmosphere, the level of the medium was lowered to culture cells on ALI, and the medium was changed every 2–3 days. Accordingly, transepithelial electrical resistance (TEER) was measured with am EVOM2 equipped with an STX3 electrode (World Precision Instruments, LLC, Hertfordshire, U.K.), and the baseline was corrected using empty inserts. Differentiation was carried out until increased TEER stayed stable for at least two consecutive measurements, which was achieved within 9–14 days. The volumes used for the individual steps and plates are summarized in Table 2.

**Table 2:** Amount of Cell Culture Medium Used for the Differentiation of Calu-3 Cells^a^

	12-well		24-well	
chamber	basolateral	apical	basolateral	apical
cell seeding	1.2 mL	0.4 mL	0.8 mL	120 μL
air–liquid interface	0.4 mL		250 μL	
TEER measurement	1.5 mL	1 mL	0.8 mL	0.4 mL

a Abbreviation: TEER, transepithelial electrical resistance.

### Peptide Stability

Peptide stability was accessed as recently reported on differentiated Calu-3 cells in translucent 24-well Thincert inserts.^36^ As described for the permeability assay, cells were washed with assay buffer, and 120 μL of the peptide (diluted in assay buffer) was added to the apical compartment, while 250 μL of fresh assay buffer was kept in the basolateral chamber. At indicated time points, 100 μL of apical peptide solution was collected and precipitated in 200 μL of ACN/ethanol (1:1, v/v, PanReac AppliChem, part of ITW, Chicago Illinois) for at least 12 h at −20 °C. After 5 min centrifugation at 12,000*g*, the supernatant was transferred to a Spin-X Tube (Costar Spin-X Centrifuge Tube, 0.22 μm, Corning, Corning, New York) and centrifuged for 1 h at 12,000*g*. The filtrate was mixed 1:1 with H_2_O and analyzed by analytical RP-HPLC on a VariTide RPC column (250 mm × 4.6 mm, 200 Å, 6 μm, Agilent Technologies, Santa Clara, California,). The amount of eluent B in eluent A was gradually increased by 1.25%/min at a flow rate of 1 mL/min, and eluents were tracked by fluorescence (λ_ex_ = 525, λ_em_ = 572 nm). The amount of intact peptide was calculated from the relative area under the curve integrated with OpenLab EZChrom (Agilent). Eluents were collected and identified by MALDI-ToF mass spectrometry.

### Permeability Assay

Apparent permeability (*P*_app_) was measured on differentiated Calu-3 cells seeded on translucent 12-well Thincert inserts. Directly before and after the permeability assay, TEER measurements were performed, and data was excluded in the case of a drop of TEER. Further, cells were not used for the permeability setup if TEER was less than 300 Ω cm^2^ over empty inserts.^28,40,41^

As reference samples, fluorescein sodium (Fl, Carl Roth) and fluorescein isothiocyanate-dextran (average mass of 4000 Da, FD4, Sigma-Aldrich) were used. Both compartments were washed twice with assay buffer, which consists of DMEM/F-12 and Phenol Red-free (Gibco) supplemented with 0.05% (*m*/*v*) Casein (Sigma-Aldrich), and cells were adjusted in this buffer for 30 min under a humidified atmosphere. Peptides were diluted from 30 mM DMSO stocks and other compounds from 30 mM aqueous stocks to 31.6 μM in assay buffer (with ∼1% DMSO, v/v). Cells were stimulated with 400 μL of compound solution in the apical chamber and 1.2 mL of fresh assay buffer in the basolateral chamber. At 0, 6, and 24 h, 300 μL of samples was collected from the basolateral chamber and immediately replaced with fresh buffer. Together with a dilution series of the tested compounds, all samples were transferred to a black 96-well plate (BRAND GmbH, Wertheim, Germany), and either Tam fluorescence (λ_ex_ = 542 ± 20 nm, λ_em_ = 587 ± 20 nm) or fluorescein/fluorescein isothiocyanate (FITC) fluorescence (λ_ex_ = 492 ± 10 nm, λ_em_ = 517 ± 10 nm) was measured using a Tecan Spark. Linear regression of concentration-fluorescence signals was performed using Prism to calculate the concentration of samples. Finally, the apparent permeability was calculated according to the following equation:

$$P_{\mathrm{app}}=\frac{\frac{\Delta n}{\Delta t}}{A\cdot c_{\mathrm{apical}}}$$

where *P*_app_ is the apparent permeability [cm/s],

$\frac{\Delta n}{\Delta t}$ is the flux [mol/s],

*A* is the area [cm^2^], and

*c*_apical_ is the concentration of the apical solution [mol/cm^3^].

### Intranasal Administration of Peptides and Tissue Preparation

Male C57BL/6N mice aged 8 weeks were housed in groups of 2–5 per cage in accordance with 2010/63/EU and Society of Laboratory Animal Science (GV SOLAS) guidelines in a climate-controlled room under standard conditions at room temperature of 21 ± 2 °C, relative humidity 55 ± 15%, and regular 12 h light–dark cycles. During the experiment, animals were placed individually in cages with free access to food and water. They were monitored daily for their general health and well-being. All experimental procedures were evaluated and accepted by the animal experimental committee and approved by the local authorities (TVV57/20). Peptides were prepared in chloride form, as described previously,^32^ and administrated at a concentration equivalent to ten times the EC_50_ in DPBS (Gibco). Mice were briefly anesthetized using 2.5% isoflurane inhalation anesthesia (Baxter, Deerfield, Illinois).

The anesthetized animal was held in a supine position at a ∼45° angle to optimize intranasal absorption and minimize loss of the administered solution due to gravity. To ensure noninvasive delivery and prevent potential mucosal damage, peptides were administered dropwise directly onto the external nasal openings of anesthetized mice using a 10 μL Eppendorf micropippete (Merck KgaA, Darmstadt, Germany), allowing natural inhalation. Since no physical insertion was performed, the risk of mechanical trauma was minimized. Animals were euthanized at 15 min, 1, 2, 6 h, or 24 h after intranasal peptide administration. An intraperitoneal injection of a xylazine/ketamine cocktail (0.25 mg of xylazine, Elanco GmbH, Cuxhaven, Germany, and 2.5 mg of ketamine, bela-pharm GmbH, Vechta, Germany, per 25 g of mouse) was administrated under 2.5% isoflurane inhalation anesthesia (Baxter). The chest cavity was opened, and a cardiac puncture was performed. Mice were transcardially perfused with DPBS (Gibco) until the intravascular compartment of the brain was cleared of blood cells. To dissect the brain, the mandible and maxilla were removed to improve access. The skull was incised along the sagittal suture, and the cranial bones were carefully detached. The orbital bones were then precisely extracted. Special attention was given to preserving the integrity of the brain, ensuring that the olfactory bulb remained intact during removal to avoid mechanical loss of the administered peptide. The tissue was fixed using 4% paraformaldehyde (PFA, Carl Roth) for a 24 h period. Following that, brains immersed in a series of sucrose solutions (Carl Roth) with concentrations of 10, 20, and 30% (v/v) were employed for 24 h each at 4 °C. Next, the brain tissues were frozen at −20 °C and sectioned sagittally to a thickness of 20 μm using a cryostat at −24 °C (Leica Microsystems, Wetzler, Germany).

### Immunohistochemistry Staining and Evaluation of Specific Signals in Brain Sections

To prepare the tissues for immunostaining, they were blocked using 10% (*m/v*) normal goat serum (NGS, Jackson Immunoresearch Europe Ltd., Ely, U.K.) with 0.3% (v/v) Triton (Roche Diagnostics, Mannheim, Germany) diluted in DPBS (Gibco) for 1 h. Next, the tissues were incubated in rabbit anti-Tam antibody (TRITC polyclonal antibody, Invitrogen, Thermo Fisher) diluted 1:200 in DPBS overnight. After five-times 5 min washes with DPBS, tissues were stained with secondary goat antirabbit antibody and labeled with Alexa Fluor 633 (Thermo Fisher) diluted 1:500 in DPBS for 1 h. Finally, tissues were washed 5 times in DPBS for 5 min each and then stained with 4,6-diamidino-2-phenylindole (DAPI, SERVA Electrophoresis) for 5 min. The tissues were washed with H_2_O and mounted on slides using a fluorescence mounting medium (Dako, Carpinteria, California). Whole-brain images were acquired by scanning on an AxioScan (Carl Zeiss), and confocal microscopy was carried out on an Olympus FV1000 (Olympus Corporation, Tokyo, Japan). The presence of specific signals in two regions of each olfactory bulb, the hypothalamus, and the cortex region in the brain sections was more closely evaluated using Netscope software (Net-Base GmbH, Freiburg, Germany). Specific signals were counted as showing up in the Tam channel (λ_ex_ = 548, λ_em_ = 561 nm) and anti-Tam channel (λ_ex_ = 650, λ_em_ = 673 nm) but not in the empty channel (λ_ex_ = 488, λ_em_ = 509 nm). Data were visualized and tested with ANOVA and posthoc tests using Prism (Version 9.0).

## Results

### Fluorescence Labeling of Peptides Selective for Neuropeptide Y_1_ and Y_2_ Receptors

Peptides were synthesized by SPPS and purified to ≥95%, as demonstrated in two different HPLC setups. The identity of the compounds was confirmed by mass spectrometry (Supporting Information Table S1).

Receptor activity was tested on the Y_1_R and Y_2_R using COS-7 cells stably expressing the receptors and the chimeric G protein Gα_Δ6qi4myr_ (kindly provided by Evi Kostenis). Concentration–response curves are shown in Figure 1, and the activity data are summarized in Table 3. Wild-type pNPY (compound **1**) activates the Y_1_R with low nanomolar potency (EC_50_ = 1.1 nM) and with subnanomolar potency for the Y_2_R (EC_50_ = 0.1 nM). The Tam-labeled variant of pNPY (compound **2**) shows full receptor efficacy and similar receptor potencies to pNPY with EC_50_ values of 0.8 and 0.3 nM for the Y_1_R and Y_2_R, respectively.

**Figure 1:**
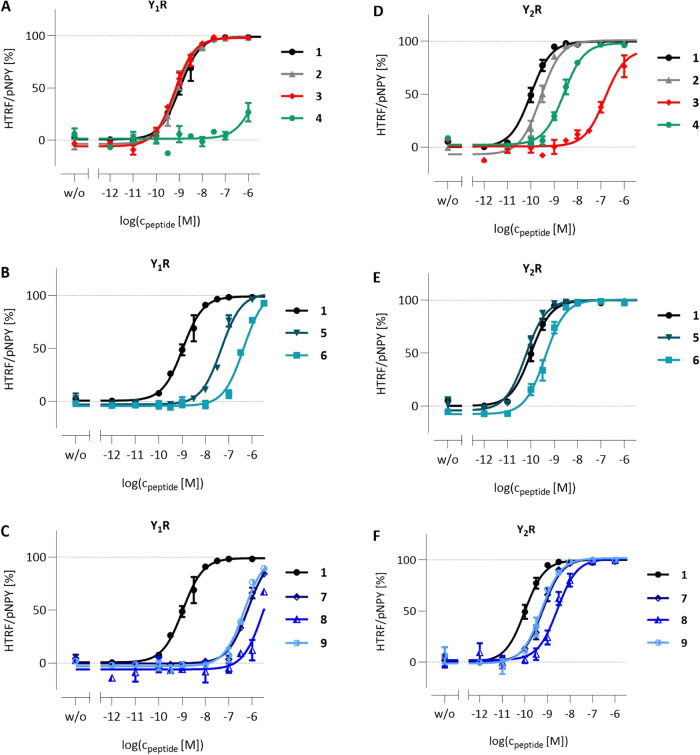
Concentration–response curves of peptides on the Y_1_R and Y_2_R. The activity was monitored by IP_1_ accumulation in COS-7 cells stably transfected with the hY_1_R (A–C) or hY_2_R (D–F) and chimeric G protein. (A, D) NPY derivatives, (B, E) PYY derivatives without lipidation, and (C, F) lipidated PYY analogues. Data are shown as mean ± SEM, *n* ≥ 2. Abbreviations: HTRF, homogeneous time-resolved fluorescence; IP_1_, inositol monophosphate; pNPY, porcine neuropeptide Y; PYY, peptide YY; SEM, standard error of the mean; Y_1_R, neuropeptide Y_1_ receptor; Y_2_R, neuropeptide Y_2_ receptor.

**Table 3:** Activity of Peptides on the Y_1_R and Y_2_R^a^

		Y_1_R			Y_2_R		
	peptide	EC_50_ [nM]	pEC_50_ ± SEM	*E*_max_ ± SEM	EC_50_ [nM]	pEC_50_ ± SEM	*E*_max_ ± SEM
1	pNPY	1.1	8.97 ± 0.04	99 ± 2	0.1	10.00 ± 0.05	100 ± 1
2	[K^4^(Tam)]-pNPY	0.8	9.11 ± 0.07	99 ± 3	0.3	9.58 ± 0.06	101 ± 2
3	[K^4^(Tam), F^7^, P^34^]-pNPY	0.6	9.21 ± 0.05	98 ± 2	>100	n. d.	n. d.
4	[K^4^(Tam), Ahx^5–24^]-NPY	>100	n. d.	n. d.	2.6	8.59 ± 0.06	98 ± 2
5	PYY_3–36_	47	7.33 ± 0.06	102 ± 3	0.06	10.22 ± 0.06	100 ± 2
6	[K^4^(Tam)]-PYY_3–36_	>100	n. d.	n. d.	0.4	9.40 ± 0.06	100 ± 2
7	[K^4^(Tam), K^7^(γGlu-Pam)]-PYY_3–36_	>100	n. d.	n. d.	0.6	9.21 ± 0.05	101 ± 2
8	[K^4^(Tam), K^7^ (γGlu-Odd)]-PYY_3–36_	>100	n. d.	n. d.	2.8	8.56 ± 0.09	101 ± 4
9	[K^4^(Tam), K^7^ (γGlu-C18)]-PYY_3–36_	>100	n. d.	n. d.	0.5	9.27 ± 0.07	102 ± 3

a The IP_1_ Accumulation Assay was Carried Out with COS-7 Cells Stably Transfected with the hY_1_R/hY_2_R and Chimeric G Protein *n* ≥ 2. Abbreviations: Ahx, 6-aminohexanoic acid; EC_50_, half-maximal efficient concentration; *E*_max_, maximal effect; IP_1_, inositol monophosphate; Pam, palmitic acid; Odd, octadecanonic diacid; pNPY, porcine neuropeptide Y; PYY, peptide YY; SEM, standard error of the mean; Tam, 6-carboxytetramethylrhodamine; Y_1_R, neuropeptide Y_1_ receptor; Y_2_R, neuropeptide Y_2_ receptor.

[K^4^(Tam), F^7^, and P^34^]-pNPY (compound **3**) maintains pNPY-like activation at the Y_1_R but a high loss of activity at the Y_2_R. Peptide **4** shows no significant activity at the Y_1_R and full efficacy but a 26-fold loss in EC_50_ at the Y_2_R. In contrast, PYY_3–36_ (compound **5**) has equipotent activity at the Y_2_R as pNPY and nearly 800-fold selectivity for Y_2_R compared to Y_1_R. Variants of PYY_3–36_ with a fluorescent label at position Lys^4^ (compound **6**) show some loss of Y_2_R activity (EC_50_ = 0.4 nM). Further modification with replacement of Ala^7^ by a lysine linked to γ-Glu and subsequently C16 palmitic acid (Pam) lead to compounds with no further loss of activity (peptide **7**, EC_50_ = 0.6 nM at Y_2_R). In contrast, peptide **8**, with attachment of the C18 octadecanonic diacid (Odd), displays reduced Y_2_R activity with EC_50_ = 2.8 nM. Peptide **9**, which contains C18 acid, is equipotent as palmitoylated compound **7**. Compounds **6**–**9** only activate the Y_1_R at high concentrations, showing high selectivity for the Y_2_R for all PYY_3–36_ analogues.

In summary, pNPY (peptide 1) and its fluorescently labeled variant (peptide 2) are potent ligands at both receptors, Y_1_R and Y_2_R. Peptide 3 shows excellent and selective Y_1_R activation, while peptides 4–9 are Y_2_R-selective with sub- to low nanomolar Y_2_R potency and excellent selectivity over the Y_1_R.

### Peptide Stability in the Calu-3 Supernatant

To assess the peptide stability, samples incubated in the Calu-3 supernatant were analyzed for degradation over 48 h (Figure 2). Compound **2** shows significant degradation (42 ± 5%) within 24 h, while the Y_1_R selective compound **3** is more stable, with only 24 ± 8% degradation after 24 h. MALDI-ToF analysis of the major degradation products reveals cleavage of a single Tyr from either the C- or N-terminus. In contrast, [K^4^(Tam), Ahx^5–24^]-pNPY rapidly degrades, with a half-life of 1 h. As early as 6 h after stimulation, almost no intact compound **4** is detectable. The main degradation products of peptide **4** are YPSK­(Tam)-Ahx, YPSK­(Tam)-Ahx-R, and PSK­(Tam)-Ahx after 24 h. The fluorescently labeled PYY_3–36_ variant (compound **6**) shows a greater stability than peptide **3**, with a first shoulder in RP-HPLC at 48 h that has been identified as fragment 3–34. The lipidated PYY_3–36_ analogues **7**–**9** are the most stable compounds, with less than 10% degradation over 48 h.

**Figure 2:**
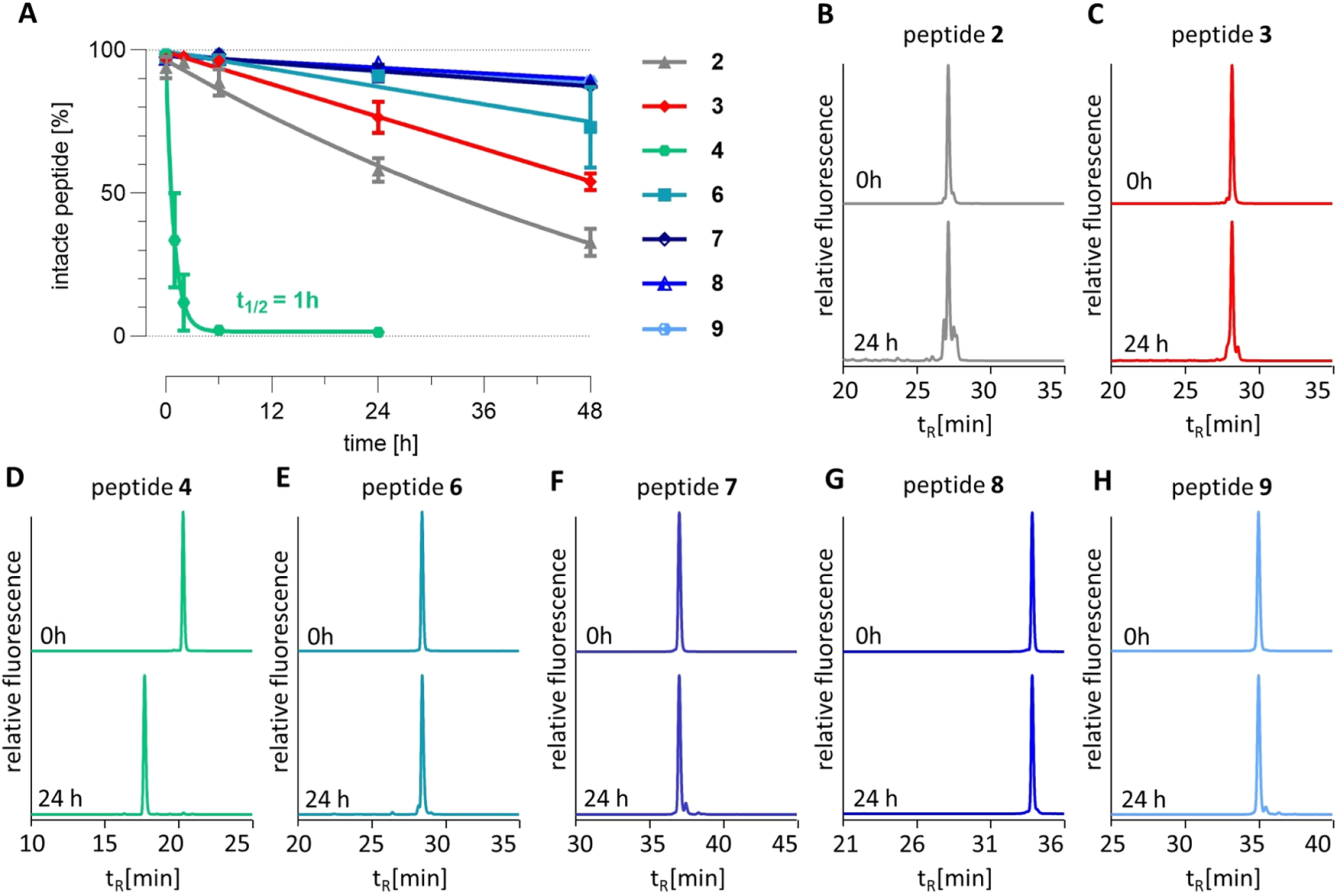
Stability of fluorescently labeled peptides in the Calu-3 cell supernatant. Peptide solutions were incubated in the apical chamber on differentiated Calu-3 cells for up to 48 h, and peptide degradation was monitored by using the fluorescent channel on RP-HPLC. Eluent B (ACN + 0.08% TFA) in eluent A (H_2_O + 0.1% TFA) was increased by 1.25%/min at 40 °C on a VariTide RPC column (250 mm × 4.6 mm, 200 Å, 6 μm, Agilent Technologies). The amount of intact peptide (A) was calculated from the relative area under the curve and is shown as mean ± SEM, *n* = 2. Gradients were either 10–60% eluent (A) in (B) (B–D, H) or 5–70% eluent (B) in (A) (E–G). Abbreviations: ACN, acetonitrile; RP-HPLC, reversed-phase high-performance liquid chromatography; TFA, trifluoroacetic acid; *t*_R_, retention time; *t*_1/2_, half-life.

### Permeability of Fluorescently Labeled Peptides on Calu-3 Cells

Calu-3 cells were used as a model system of the respiratory epithelium. Prior to the permeability assay, cell differentiation was monitored by an increase of TEER by 400–800 Ω cm^2^. Permeability was measured by the translocation of fluorescently labeled compounds from the apical chamber to the basolateral compartment over 24 h. Fl and FD4 are used as controls, and *P*_app_ was determined as 115 ± 11 × 10^–9^ and 34 ± 10 × 10^–9^ cm/s, respectively (Figure 3). Most peptides (**2**, **3**, **6**, **7**, **9**) showed similar *P*_app_ values, ranging from 12 ± 1 × 10^–9^ cm/s to 26 ± 4 × 10^–9^ cm/s for peptides **9** and **6**, respectively. Peptide **8** exhibited a slightly increased *P*_app_ of 43 ± 8 × 10^–9^ cm/s. In contrast, compound **4** was determined more in the range of Fl with *P*_app_ = 88 ± 4 × 10^–9^ cm/s.

**Figure 3:**
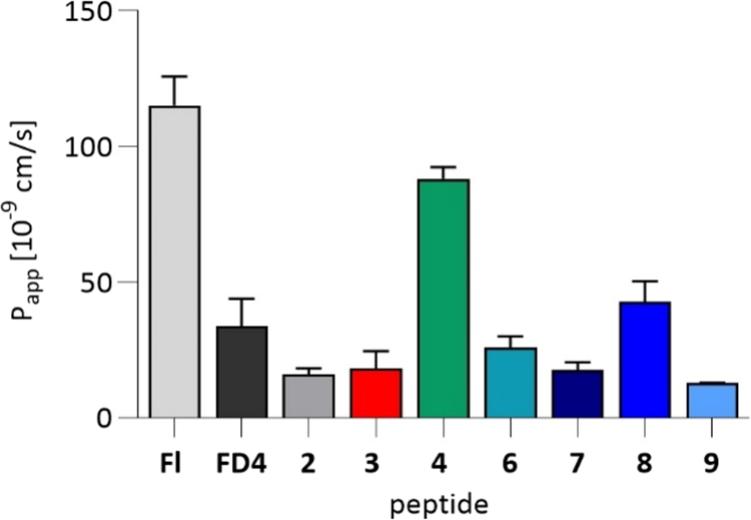
Permeability of fluorescently labeled peptides tested on Calu-3 cells. Data are displayed as mean ± SEM, *n* ≥ 2. Abbreviations: ALI, air–liquid interface; FL, fluorescein sodium; FD4, fluorescein isothiocyanate-dextran, average mass 4 kDa; *P*_app_, apparent permeability; TEER, transepithelial electrical resistance.

### Transport of Bioactive Peptides through Calu-3 Cells Measured by the Combined Permeability and Receptor Activity Assay

The amount of active compounds translocated across Calu-3 cells was measured by combining the permeability assay with a subsequent activity assay (Figure 4). Therefore, dilution series from the permeability assay setups were used to stimulate COS-7_hY_1_R_Gα_Δ6qi4myr_ or COS-7_hY_2_R_Gα_Δ6qi4myr_ in an IP_1_ accumulation assay to determine the concentration–response curve (displayed on the right *X*-axis, Figure 4B). In parallel, samples from the basolateral compartment of Calu-3 cells, either stimulated apically with peptide or buffer, are included (left side of the graph, Figure 4B). The samples from unstimulated Calu-3 cells were used as a negative control. These samples did not induce IP_1_ accumulation in the stably transfected COS-7 cells, but the signals showed a slight decrease the longer the buffer was preincubated over the Calu-3 cells.

**Figure 4:**
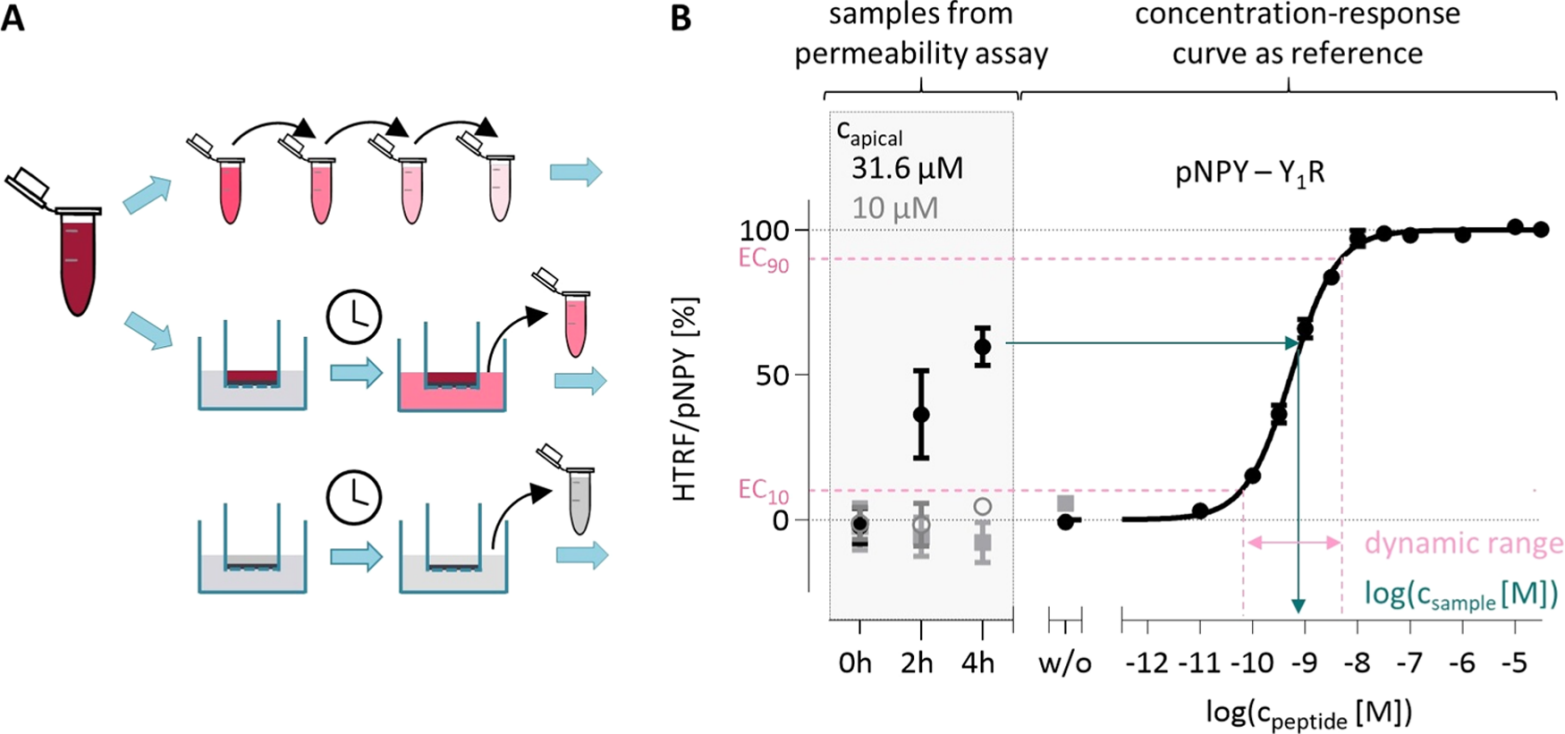
Setup of the combined Calu-3 permeability and IP1 accumulation assay. (A) Assay setup: the stock solution of the peptide was diluted and either directly used to measure concentration–response curves or transferred to the apical compartment of differentiated Calu-3 cells. Samples from the basolateral compartment of differentiated Calu-3 cells—either incubated with peptide solution or buffer for negative control (shown in gray)—were tested in activity assays. (B) IP_1_ accumulation assay with samples from permeability assays and concentration–response curves, plotted as mean ± SEM, *n* ≥ 2. EC_10_ and EC_90_ determine the dynamic range of the concentration–response curves (pink lines). Within this range, relative HTRF can be aligned to the concentration of the active compound in the sample (teal arrows). Abbreviations: EC_10_, 10% effective concentration; EC_90_, 90% effective concentration; HTRF, homogeneous time-resolved fluorescence; IP_1_, inositol monophosphate; pNPY, porcine neuropeptide Y; SEM, standard error of the mean; Y_1_R, neuropeptide Y_1_ receptor.

Within the range of 10 and 90% effective concentrations (EC_10_ and EC_90_, respectively), the HTRF signal is almost directly proportional to the logarithmic concentration of the active compound. Thus, the concentration of the samples can be calculated within the dynamic range or can be determined to be less than or more comparable to that of EC_10_ or EC_90_, respectively (Table 4). The data used for these calculations are shown in the Supporting Information (Figure S1).

**Table 4:** Concentrations of Bioactive Peptides Translocated through Calu-3 Cells, as Calculated from the Combined Permeability and Activity Assay^a^

		dynamic range concentration	calculated concentration in the Calu-3 permeability assay log(*s*_ample_ [M])			
			10 μM apical		31.6 μM apical	
receptor	peptide	log(*c* [M])	2 h	4 h	2 h	4 h
Y_1_R	pNPY (peptide **1**)	0.06 nM – 4.90 nM	<0.06 nM	<0.06 nM	0.29 nM	0.81 nM
		−10.22 – −8.31	<−10.22	<−10.22	−9.54 ± 0.44	−9.09 ± 0.17
Y_1_R	[F^7^, P^34^]-pNPY	0.04 nM – 2.82 nM	0.09 nM	0.43 nM	1.90 nM	>2.82 nM
		−10.44 – −8.55	−10.03 ± 0.42	−9.36 ± 0.23	−8.72 ± 0.10	>−8.55
Y_1_R	[K^4^(Tam), F^7^, P^34^]-pNPY (peptide **3**)	0.02 nM – 2.70 nM	0.05 nM	0.27 nM	1.06 nM	>2.70 nM
		−10.67 – −8.57	−10.27 ± 0.45	−9.58 ± 0.28	−8.98 ± 0.05	>−8.57
Y_2_R	[K^4^(Tam), Ahx^5–24^]-NPY (peptide **4**)	0.21 nM – 17.0 nM	<0.21 nM	<0.21 nM	<0.21	<0.21
		−9.67 – −7.77	<−9.67	<−9.67	<−9.67	<−9.67
Y_2_R	PYY_3–36_ (peptide **5**)	0.01 nM – 0.54 nM	>0.54 nM	>0.54 nM	>0.54 nM	>0.54 nM
		−11.21 – −9.27	>−9.2	>−9.27	>−9.27	>−9.27
Y_2_R	[K^4^(Tam), K^7^(γGlu-Pam)]-PYY_3–36_ (peptide **9**)	0.07 nM – 2.26 nM	0.08 nM	0.12 nM	0.35 nM	0.84 nM
		−10.18 – −8.65	−10.08 ± 0.03	−9.93 ± 0.12	−9.45 ± 0.02	−9.07 ± 0.04

a Samples from different peptides were tested to activate the Y_1_R or Y_2_R. The dynamic range was calculated from EC_10_ and EC_90_. The concentration of the active compound in the basolateral chamber was calculated after 2 and 4 h from HTRF signals compared to the concentration–response curve of the individual peptide. Two different concentrations of each peptide were tested in the permeability assay. Values are given as absolute concentration and log­(c [M]) ± SEM, *n* ≥ 2. Abbreviations: Ahx, 6-aminohexanoic acid; EC_10_, 10% effective concentration; EC_90_, 90% effective concentration; HTRF, homogeneous time-resolved fluorescence; Pam, palmitic acid; Odd, octadecanonic diacid; pNPY, porcine neuropeptide Y; PYY, peptide YY; SEM, standard error of the mean; Tam, 6-carboxytetramethylrhodamine; Y_1_R, neuropeptide Y_1_ receptor; Y_2_R, neuropeptide Y_2_ receptor.

The dynamic range for all tested peptides was in the picomolar to nanomolar concentration range. For samples within this range, the amount of bioactive peptide translocated across the Calu-3 cell layer increased in a time- and concentration-dependent manner. Compared to pNPY, samples of [F^7^, P^34^]-pNPY showed translocation of more bioactive compounds. The concentrations of [K^4^(Tam), F^7^, P^34^]-pNPY were very much in the same range as the nonfluorescent analogue of this compound. For [K^4^(Tam), Ahx5–24]-NPY, no active compound measurably penetrated the Calu-3 cell layer, while for PYY_3–36_, all samples exceeded the dynamic range of this peptide, with concentrations higher than 0.54 nM. For [K^4^(Tam), K^7^(γGlu-Pam)]-PYY_3–36_, a concentration- and time-dependent increase of the bioactive compound was observed, but the concentrations are lower than for the two Y_1_R-selective peptides.

### Detection of Fluorescently Labeled Peptides after Intranasal Application *In Vivo*

Peptides showing the best properties with respect to activity, selectivity, stability, and permeability in the *in vitro* characterization were selected for *in vivo* studies. Due to its good activity, stability, and permeability, [K^4^(Tam), F^7^, P^34^]-pNPY (peptide **3**) was used. Of the Y_2_R-selective compounds, the three lipidated peptides showed the best stability. [K^4^(Tam), K^7^(γGlu-Pam)]-PYY_3–36_ (peptide **7**) was chosen because of its higher Y_2_R potency than [K^4^(Tam), K^7^(γGlu-Odd)]-PYY_3–36_ and its higher permeability than [K^4^(Tam), K^7^(γGlu-C18)]-PYY_3–36_. Peptides were applied intranasally in mice, and brain tissue samples were obtained for analysis 15 min to 24 h after intranasal (IN) application. Additionally, pNPY and PYY were used as a negative control.

Within the brain section, the olfactory bulb (OB), the hypothalamus, and the cortical region between the two brain regions were evaluated by using two imaging techniques (Figure 5). The slide scanner allows examination of the whole-brain section to localize the desired areas and zoom in for detailed analysis. In contrast, confocal microscopy required preselection of the area to be imaged but provided an improved signal-to-noise ratio (Figures 6 and 7). Both methods enabled the detection of specific signals as vesicular-like structures in close proximity to the DAPI-labeled cell nuclei.

**Figure 5:**
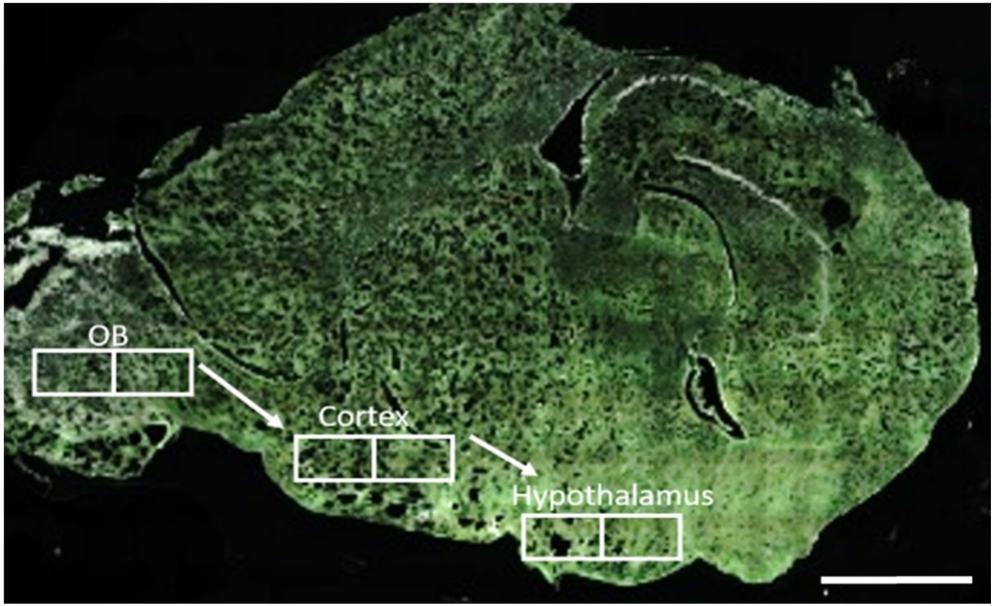
Representative scan of the mouse brain with areas used for quantification of fluorescence signals. Full-section scans were obtained with AxioScan. Scale bar: 1 mm. Abbreviation: OB, olfactory bulb.

**Figure 6:**
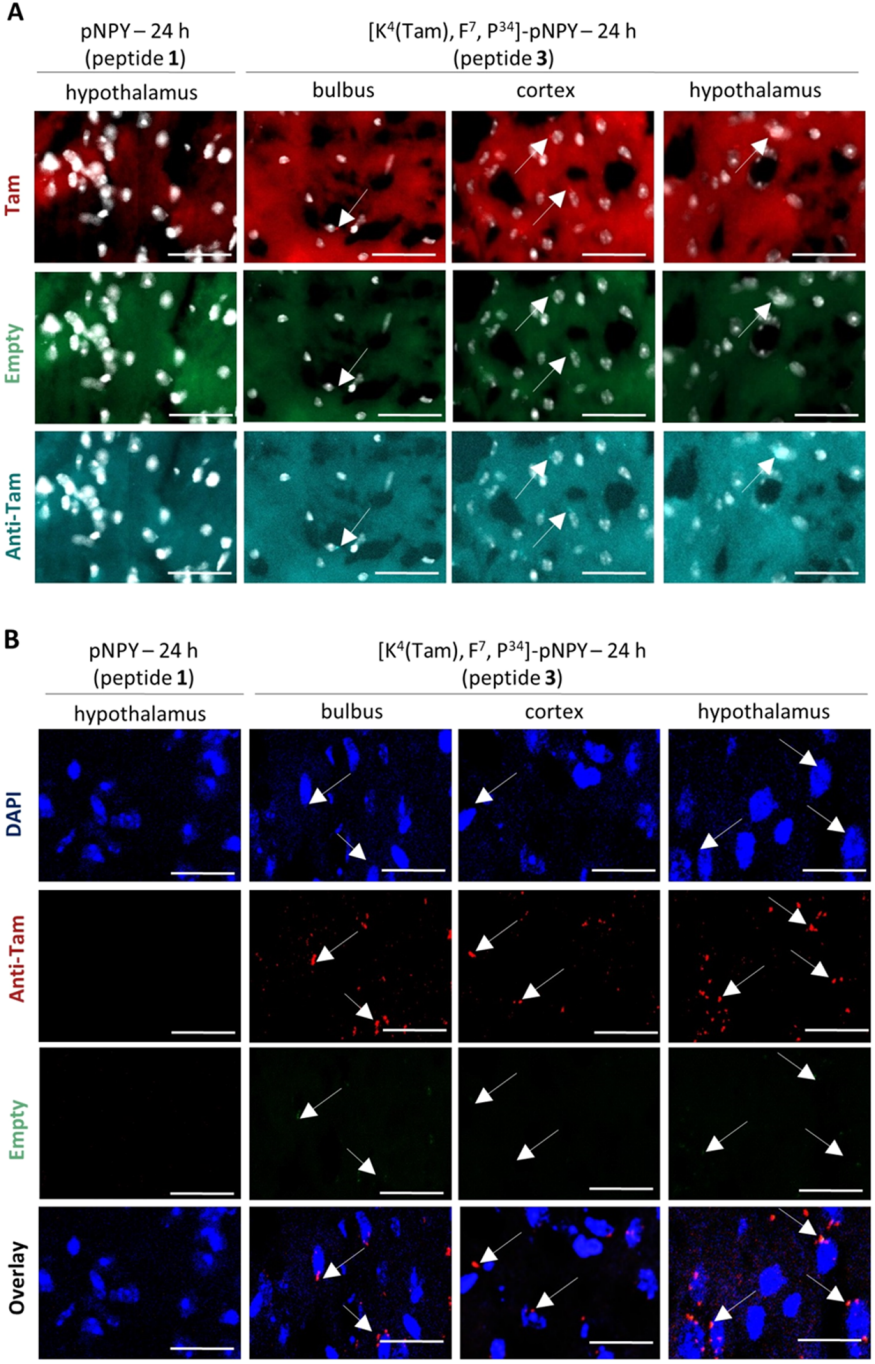
Comparison of AxioScan and confocal microscopy for the detection of the Tam-labeled Y_1_R agonist *in vivo*. (A) Images from AxioScan, with nuclei from DAPI staining in white, Tam fluorescence in red, anti-Tam antibody labeling in turquoise, and empty channels for determination of background fluorescence are in green. Scale bar: 50 μm. (B) Images from confocal microscopy, nuclei from DAPI staining in blue, anti-Tam antibody labeling in red, and empty channels for determination of background fluorescence in green. Scale bar: 10 μm. All images are from the brain section 24 h after nasal application and are representative of three independent experiments. Abbreviations: DAPI, 4,6-diamidino-2-phenylindole; pNPY, porcine neuropeptide Y; Tam, 6-carboxy-tetramethylrhodamine.

**Figure 7:**
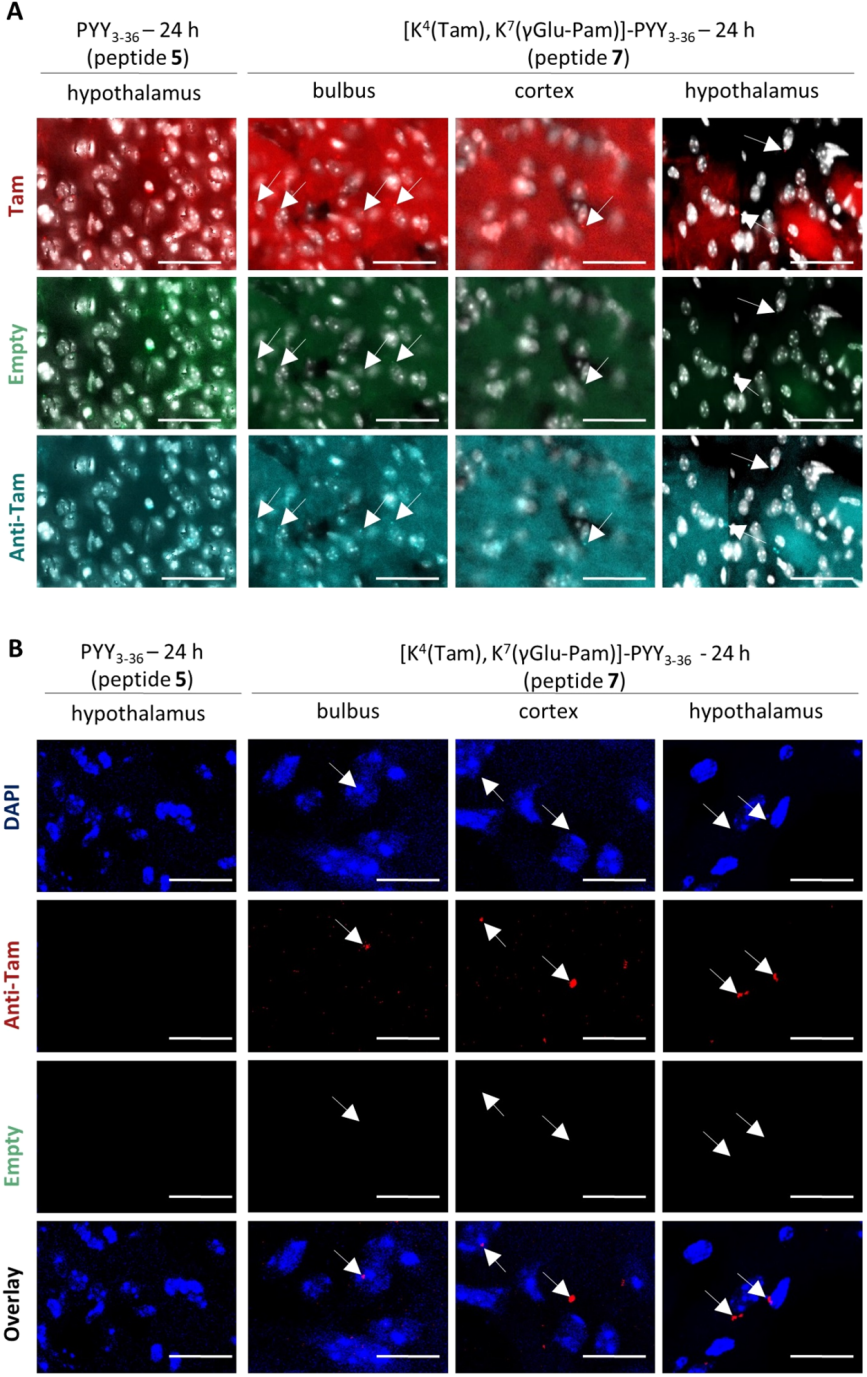
Comparison of AxioScan and confocal microscopy for the detection of the Tam-labeled Y_2_R agonist *in vivo*. (A) Images from AxioScan, with nuclei from DAPI staining shown in white, Tam fluorescence in red, anti-Tam antibody labeling in turquoise, and empty channes for determination of background fluorescence in green. Scale bar = 50 μm. (B) Images from confocal microscopy, with nuclei from DAPI staining displayed in blue, anti-Tam antibody labeling in red, and empty channels for determination of background fluorescence in green. Scale bar = 10 μm. All images are from the brain section 24 h after nasal application and are representative of three independent experiments. Abbreviations: DAPI, 4,6-diamidino-2-phenylindole; Pam, palmitic acid; PYY, peptide YY; Tam, 6-carboxy-tetramethylrhodamine.

To distinguish between unspecific signals from brain tissue and those from Tam-labeled peptides, an empty channel (green fluorescent protein channel) and immunohistochemical staining with an anti-Tam primary antibody in combination with a secondary antibody labeled with an Alexa Fluor 633 ligand were included. Signals from the Tam channel, which also appear in the empty channel, were rated as background fluorescence and not counted. In contrast, signals in both the Tam channel (from direct fluorescent labeling of the peptide) and the anti-Tam channel (from immunostaining) were validated as specific.

### Quantification of Specific Signals *In Vivo*

The specific signals from peptides **3** and **7** were quantified for different time points in the inspected areas (Figure 8). While specific Tam signals can be detected in all regions at all tested time points, a minor amount of background signals is observed in the brain sections from mice treated with unlabeled peptides pNPY and PYY, marked as the negative control (nc). For mice treated with the [K^4^(Tam), F^7^, P^34^]-pNPY (peptide **3**), more signals were detected in the olfactory bulb and cortex at 15 min, while the hypothalamus showed the greatest number of signals at 1 h. After a signal drop at 2 h for all three regions, the number of signals increased slightly to 6 h and remained stable for up to 24 h. In mice treated with [K^4^(Tam), K^7^(γGlu-Pam)]-PYY_3–36_ (peptide **7**), the most significant number of signals was observed in all three regions at 15 min. The number of signals decreased subsequently, with a partial increase in the cortex at 2 h and in the olfactory bulb and hypothalamus 6 h after IN peptide administration.

**Figure 8:**
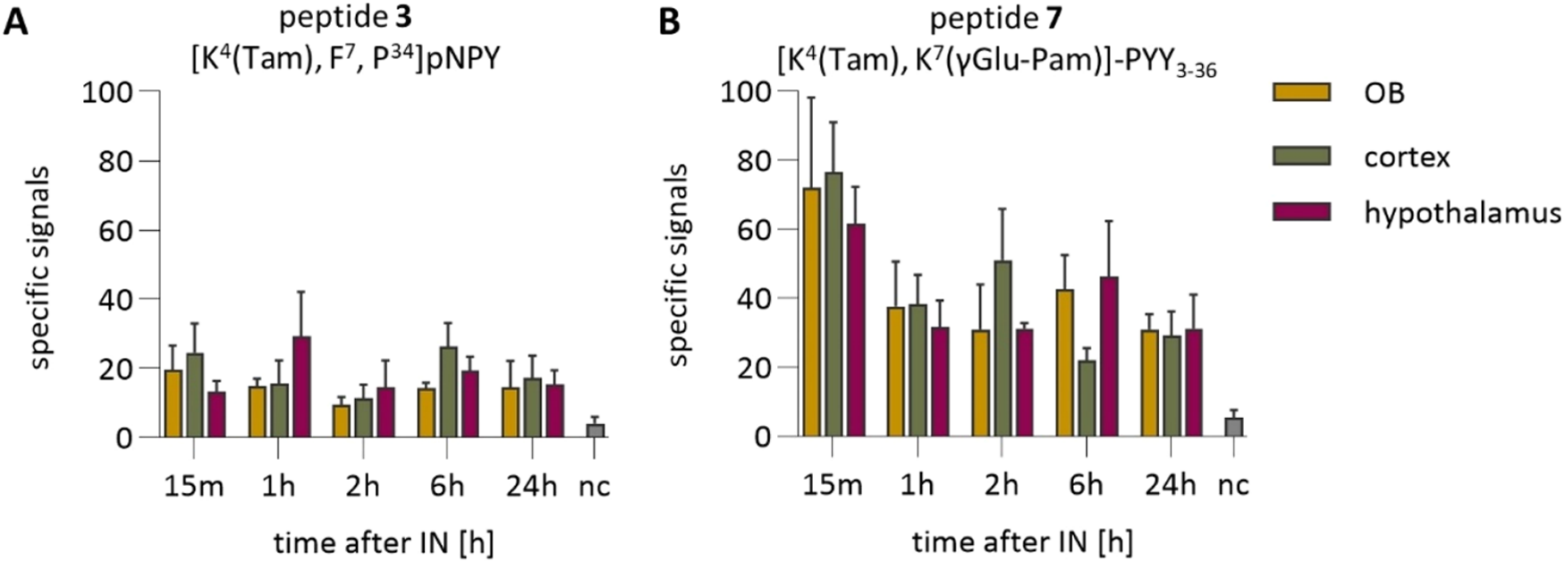
Uptake of fluorescently labeled peptides after intranasal application. Quantification of Tam-specific signals in the olfactory bulb, cortex, and hypothalamus for peptides **3** (A) and **7** (B). Data are shown as mean ± SD, *n* ≥ 3. Abbreviations: nc, negative control; OB, olfactory bulb; Pam, palmitic acid; pNPY, porcine neuropeptide Y; PYY, peptide YY; SD, standard deviation; Tam, 6-carboxytetramethylrhodamine.

## Discussion

Intranasal application of peptides selectively activating Y_1_R and Y_2_R may provide further access to the therapeutic potential of the NPY multireceptor–multiligand system. Intranasal NPY has been studied in the context of several neurological and psychiatric disorders, like post-traumatic stress disorder (PTSD).^42–46^ In animal models, intranasal application of NPY immediately after a traumatic event prevented the development of impairments in fear memories.^42^ However, the effects of intranasal NPY tested in human trials were limited. For example, a single dose of intranasal NPY showed dose-dependent, but not significant, positive trends in patients with PTSD.^44^ In the context of anxiety, Y_1_R activation is associated with reducing and Y_2_R activity is associated with promoting negative effects.^47^ Consequently, selective Y_1_R agonists may show improved anxiolytic outcomes over NPY itself, which activates both receptors with high affinity. The effect of specific neuropeptide Y receptor-targeted agonist peptides on food intake is also favored by receptor selectivity, as intravenous administration of truncated and selective PYY_3–36_ shows increased anorectic effects compared to the less selective full-length PYY.^21,24^ Again, Y_1_R and Y_2_R promote opposite effects on the appetite. The importance of receptor selectivity may contribute to the lack of effects of long-term nasal NPY on bodyweight in mice.^42^ However, nasal NPY has been found to reduce the cortical direct current potential shift associated with orexigenic effects in humans.^48^

Here, we combined the nasal application of selective Y_1_R and Y_2_R with a comprehensive pre-evaluation of compounds in cell-based assays. In addition to receptor activity and selectivity, stability and permeability were also tested on Calu-3 cells cultured as a model system of the nasal mucosa. Fluorescence labeling of the NPY derivative at Lys^4^ has been previously used for microscopic and stability assays and is well tolerated regarding receptor activity and selectivity.^36,49^ In addition, we included lipidation for PYY analogues **7**–**9**, as it is a commonly used approach to increase plasma stability.^32,50,51^ Interestingly, peptides **7**–**9** showed prolonged half-life in the serum albumin-free Calu-3 supernatant compared to nonlipidated peptide **6**.^52^ Since we have not seen any stabilizing effect of lipidation on ghrelin in the Calu-3 supernatant in a recent study, this effect seems specific to the PYY derivatives.^36^ The hydrophobic attachment may likely stabilize the secondary structure of the PYY analogues consisting of a C*-*terminal α-helix interacting with the backfolded N-terminal peptide chain, resulting in the proximity of both termini, a favorable configuration for Y receptor binding.^51^ For the permeability assay, Fl and FD4 were used as reference compounds. The *P*_app_ for Fl is in agreement with previous reports,^53–55^ while the *P*_app_ of FD4 was reported as 5-fold lower but with a significantly shorter assay time than studied here.^55^ It is likely that the transport of small molecules and larger-molecular-weight substances have different dynamics, resulting in an increased *P*_app_ for FD4 when monitored over longer time periods. Nevertheless, the permeability values from Fl and FD4 clearly show differences between the small molecule and a larger, polar compound. Most of the peptides tested showed a *P*_app_ that is comparable to that of FD4, which is also in a similar molecular weight range. The significantly increased permeability of peptide **4** can be explained by the rapid degradation of the peptide to low-molecular-weight degradation products. This is consistent with the combined permeability and activity assay design, which did not detect any bioactive peptide **4** in the basolateral chamber. By contrast, all other peptides showed sufficient stability in the Calu-3 supernatant, and bioactive compounds were able to translocate through the Calu-3 cell layer.

Based on the results of *in vitro* characterization, we selected peptides **3** and **7** to test for uptake after nasal application *in vivo* and detected Tam-specific fluorescence in the brain sections. Confocal microscopy revealed specific fluorescence signals in small, vesicle-like structures in close proximity to nuclei. pNPY and PYY are known to internalize upon interaction with their Y receptor.^56,57^ This localization could indicate that the peptides successfully interacted with their receptors after nasal application. This is also in line with the expression of Y_1_R and Y_2_R, which were found in many brain regions including the cortex and hypothalamus and the olfactory epithelium.^58–61^ However, it cannot be excluded that other internalization mechanisms may have been involved. Fatoba et al. also used fluorescence microscopy to track fluorescently labeled, truncated NPY in mice 30 min after IN administration.^46^ In their studies, fluorescence was detected from the olfactory cavity, over the cortex, to the medulla oblongata, while the hypothalamus was not examined. Again, vesicular-like structures were seen in brain regions. Unfortunately, the signals were not quantified in experiments by Fatoba et al., although the qualitative observations are in line with the data reported here.

Nose-to-brain delivery can occur along several pathways, which also determines the time it takes to reach the central nervous system (CNS). After uptake at the nasal mucosa, peptides can be transported by neuronal pathways along the olfactory or trigeminal neurons.^62,63^ In addition, compounds can enter the bloodstream from nasal tissue and cross the BBB to reach the CNS.^27^ This is less likely for NPY, as no increase in plasma levels has been reported after nasal NPY application.^64^ Within the neuronal pathway, slower intraneuronal and faster extracellular transports are distinguished. While the latter is considered to be relatively faster, intraneuronal transport includes internalization, which can take from minutes up to hours and days.^65,66^ Here, the amounts of specific signals were quantified at five different time points, from 15 min to 24 h. For both peptides, specific signals were detected 15 min after nasal application in all three examined brain regions, which could be indicative of rapid extracellular transport. Later peaks, e.g., at 1 h for peptide **3** and at 6 h for peptide **7** in the hypothalamus, could indicate additional involvement of slower intraneuronal transport.

The specific signals of peptide **3** detected in brain sections were lower than those of peptide **7**, while the Calu-3 permeability was in a range comparable to those for both peptides. However, the cell line represents only epithelial cells and therefore cannot fully capture the complexity of a multicellular tissue, especially not the role of neuronal uptake.^40^ Another reason for the reduced signals from the Y_1_R agonist (peptide **3**) could be the lower stability of the peptide, which could allow faster degradation of the peptide, followed by washout of the fluorophore. Alternatively, the reduced cellular internalization of peptide **3** compared to peptide **7** could explain the reduced number of signals. The number of signals may not correlate with the total amount of peptide as the free peptide is not detected in low concentrations. However, biochemical quantification of the peptide in brain tissue was hampered by the low dose used here in combination with PFA fixation.

Peptides **3** and **7** are effectively taken up from nasal application and are transported to the brain, where specific signals lasted for the whole examined time period of 24 h after nasal application. Also, our data underline the high potential of the peptides based on high receptor activity and selectivity, stability, and sufficient nose-to-brain delivery; it remains to be tested whether intranasally applied analogues of NPY and PYY will show improved pharmacological profiles.

## Conclusions

Nose-to-brain delivery is a highly promising approach for the delivery of drugs to cerebral targets. Peptides of the neuropeptide Y multireceptor–multiligand system are interesting lead structures, e.g., for modulation of food intake or treatment of psychological disorders, but high activity, selectivity, and stability are important properties to ensure. The Calu-3 permeability assay allows peptide absorption in a model system of the respiratory epithelium for stable peptides. Fluorescence labeling can be used to assess the permeability, stability, and *in vivo* uptake of peptides. Furthermore, intact analogues of NPY and PYY can efficiently cross the cellular barrier independent of the fluorescent label. Uptake studies *in vivo* demonstrated successful transport of compounds to the olfactory bulb, cortex, and hypothalamus after nasal application. Furthermore, the quantification of specific peptide signals indicates the involvement of different transport mechanisms, highlighting the complexity of nose-to-brain delivery.

## Supplementary Material



## Notes

Analytics of synthesized peptides and plotted data of the combined permeability and activity assay (PDF)

## Abbreviations

Δ*n*/Δ*t*: flux
λ_ex_: excitation wavelength
λ_em_: emission wavelength
*A*: area
ACN: acetonitrile
Ahx: 6-aminohexanoic acid
ALI: air–liquid interface
ATCC: American type culture collection
DAPI: 4,6-diamidino-2-phenylindole
DCM: dichloromethane
Dde: 1-(4,4-dimethyl-2,6-dioxocyclohex-1-ylidene)­ethyl
DIC: *N*,*N*-diisopropylcarbodiimide
DIPEA: *N*,*N*-diisopropylethylamine
DMEM: Dulbecco’s modified Eagle’s medium
DMF: *N*,*N*-dimethylformamide
DMSO: dimethyl sulfoxide
DPBS: Dulbecco’s phosphate-buffered saline
DPP-4: dipeptidyl aminopeptidase 4
EC_50_: half-maximal efficient concentration
EDT: ethane-1,2-dithiol
*E*_max_: maximal effect
ESI: electrospray ionization
FBS: fetal bovine serum
FD4: fluorescein isothiocyanate-dextran, average mass 4 kDa
FITC: fluorescein isothiocyanate
Fl: fluorescein sodium
Fmoc: fluorenylmethoxycarbonyl
FRET: Förster resonance energy transfer
h: human
HATU: hexafluorophosphate azabenzotriazole tetramethyl uranium
HBSS: Hanks’ balanced salt solution
HTRF: homogeneous time-resolved fluorescence
IN: intranasal
IP_1_: inositol monophosphate
LCC: liquid cover culture
MALDI-ToF: matrix-assisted laser desorption ionization time-of-flight
Mmt: monomethoxytrityl
nc: negative control
NPY: neuropeptide Y
OB: olfactory bulb
Odd: octadecanonic diacid
Oxyma: ethyl cyanohydroxyiminoacetate
p: porcine
Pam: palmitic acid
*P*_app_: apparent permeability
PFA: paraformaldehyde
PYY: peptide YY
RP-HPLC: reversed-phase high-performance liquid chromatography
SD: standard deviation
SEM: standard error of the mean
SPPS: solid-phase peptide synthesis
*t*_1/2_: half-life
TA: thioanisole
Tam: 6-carboxytetramethylrhodamine
*t*-Bu: *tert*-butyl
TEER: transepithelial electrical resistance
TFA: trifluoroacetic acid
TIS: triisopropyl silane
t_R_: retention time
YR: neuropeptide Y receptor
