# Supplementary material for: Intranasal Application of Peptides Modulating the Neuropeptide Y System
Source: ACS Pharmacol Transl Sci. 2025 Apr 1;8(4):1168–81. doi: 10.1021/acsptsci.5c00082 (PMC11997893; doi:10.1021/acsptsci.5c00082)
Supplement: Supplementary file 1 [file pt5c00082_si_001.pdf]

Supporting Information: Intranasal application of peptides modulating the neuropeptide Y system

Eva-Maria Jülke[a1], Benginur Özbay[b1], Marcin Nowicki[b], Sylvia Els-Heindl[a], Kerstin Immig[b], Karin Mörl[a], Ingo Bechmann[b*], Annette G. Beck-Sickinger[a*]

[a] Institute of Biochemistry, Faculty of Life Sciences, Leipzig University, Brüderstr. 34, 04103 Leipzig, Germany

[b] Institute of Anatomy, Faculty of Medicine, Leipzig University, Liebigstraße 13, 04103 Leipzig, Germany

[1] Eva-Maria Jülke and Benginur Özbay contributed equally.

* Correspondence to Prof. Dr. Annette G. Beck-Sickinger, abeck-sickinger@uni-leipzig.de, Institute of Biochemistry, Faculty of Life Sciences, Leipzig University, Brüderstr. 34, 04103 Leipzig, Germany

Co-Correspondence to Prof. Dr. Ingo Bechmann, ingo.bechmann@medizin.uni-leipzig.de, Institute of Anatomy, Faculty of Medicine, Leipzig University, Liebigstraße 13, 04103 Leipzig, Germany

Purity analytics of peptides

Table S1: Analytics of synthesized peptides. Peptides were characterized by experimental mass (M_{exp}) corresponding to the calculated monoisotopic mass (M_{mono}). Purity of ≥ 95% was confirmed using reversed phase-high performance liquid chromatography on (a) Jupiter Proteo 4 µm 90 Å LC column (250 x 4.6 mm, Phenomenex) and (b) Aeris Peptide 3.6 µm XB-C18 100 Å column (250 x 4.6 mm, Phenomenex), and gradually increasing eluent B in A from 20% to 70% in 40 min. Abbreviations: Ahx: 6-aminohexanoic acid; Pam: palmitic acid; Odd: octadecanonic diacid; pNPY: porcine neuropeptide Y; PYY: peptide YY; Tam: 6-carboxytetramethylrhodamine.

peptide	M_{mono} [Da]	M_{exp} ([M+H]$^+$)	$t_R^{(a)}$ [min]	$t_R^{(b)}$ [min]	purity [%]
pNPY	4251.12	4252.14	16.6	14.7	≥ 95
[K^4(Tam)]-pNPY	4663.27	4664.30	17.9	16.0	≥ 95
[F^7, P^{34}]-pNPY	4253.14	4254.14	17.2	15.3	≥ 95
[K^4(Tam), F^7, P^{34}]-pNPY	4665.29	4666,32	20.0	18.8	≥ 95
[K^4(Tam), Ahx$^{5\text{-}24}$]-NPY	2631.39	2632.40	12.2	19.8	≥ 95
PYY$_{3\text{-}36}$	4047.07	4048.10	13.0	10.2	≥ 95
[K^4(Tam)]-PYY$_{3\text{-}36}$	4459.21	4460.92	15.6	13.3	≥ 95
[K^4(Tam), K^7(γGlu-Pam)]-PYY$_{3\text{-}36}$	4883.54	4884.56	26.2	24.7	≥ 95
[K^4(Tam), K^7 (γGlu-Odd)]-PYY$_{3\text{-}36}$	4941.55	4942.56	20.5	17.7	≥ 95
[K^4(Tam), K^7 (γGlu-C18)]-PYY$_{3\text{-}36}$	4911.58	4912.59	25.7	23.5	≥ 95

Plotted data of combined permeability and activity assay

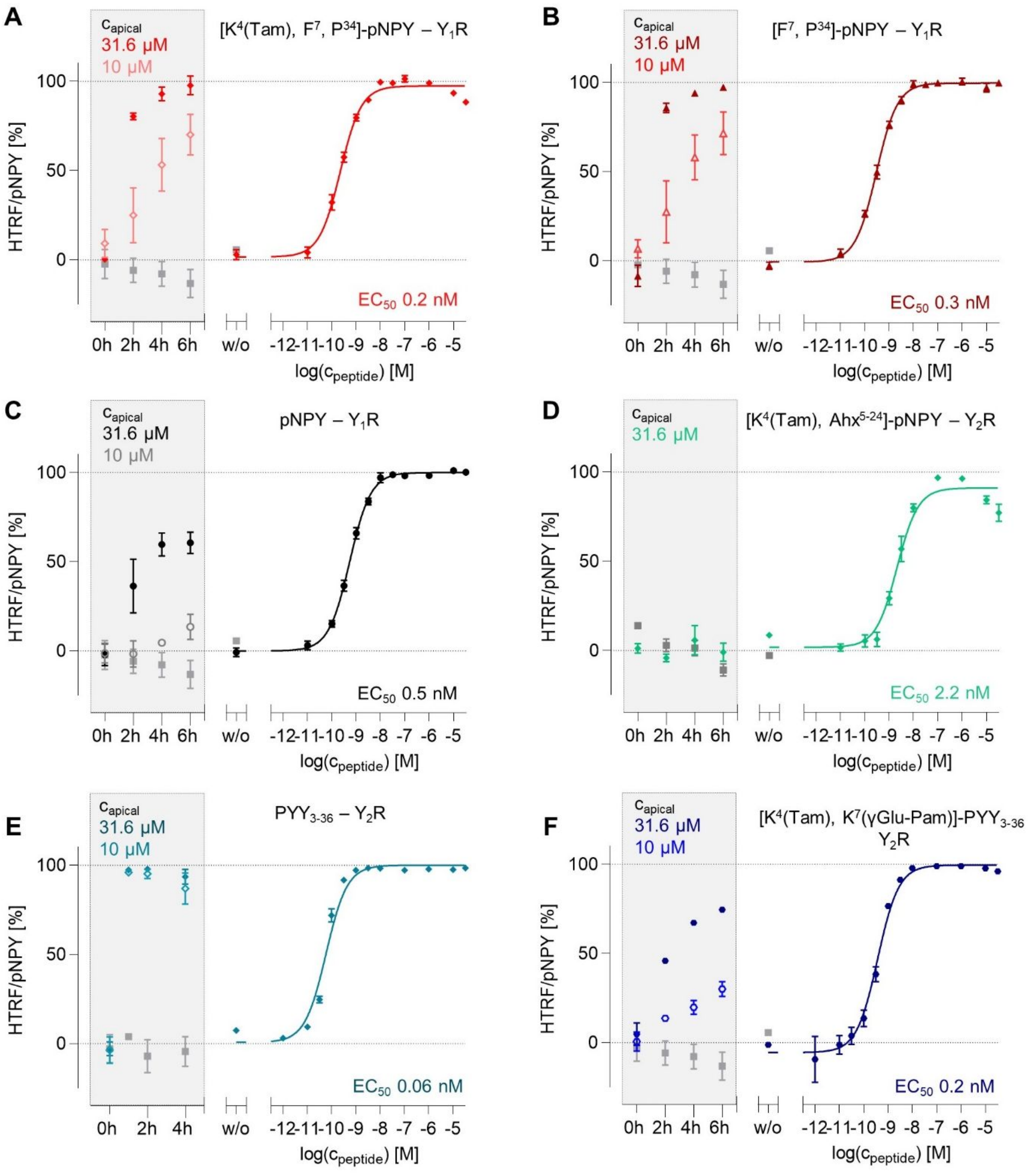

Figure S1: Concentration-response-curves and sample from combined permeability and receptor activity assay. Peptide stock solutions were diluted to measure concentration-response curves and transferred to apical compartment of differentiated Calu-3 cells. Samples from basolateral compartment of differentiated Calu-3 cells were also tested in activity assay. Activity assay was carried out with COS-7 cells stably transfected with hY_1R/hY_2R and chimeric G protein. Data are plotted as mean ± SEM. $n \geq 2$. Abbreviations: Ahx: 6-aminohexanoic acid; EC_{50}: half maximal efficient concentration; HTRF: homogeneous time-resolved fluorescence; IP_1: inositol monophosphate; Pam: palmitic acid; pNPY: porcine neuropeptide Y; PYY: peptide YY; SEM: standard error of the mean; Tam: 6-carboxytetramethylrhodamine; Y_1R: neuropeptide Y_1 receptor; Y_2R: neuropeptide Y_2 receptor.
